# Prevalence and Novel Genotypes Identification of *Enterocytozoon bieneusi* in Dairy Cattle in Yunnan Province, China

**DOI:** 10.3390/ani11113014

**Published:** 2021-10-20

**Authors:** Hai-Yang Song, Kai-Sheng Wang, Jian-Fa Yang, Hua-Ming Mao, Li-Hua Pu, Yang Zou, Jun Ma, Xing-Quan Zhu, Feng-Cai Zou, Jun-Jun He

**Affiliations:** 1 Faculty of Animal Science and Technology, Yunnan Agricultural University, Kunming 650201, China; haiyangsong125@163.com (H.-Y.S.); jsc315@163.com (J.-F.Y.); 1985039@ynau.edu.cn (H.-M.M.); 2 College of Biology and Pharmacy, Yulin Normal University, Yulin 537000, China; wks@ylu.edu.cn; 3 College of Veterinary Medicine, Yunnan Agricultural University, Kunming 650201, China; plh1995815@163.com (L.-H.P.); dreamerjm@163.com (J.M.); xingquanzhu1@hotmail.com (X.-Q.Z.); 4 State Key Laboratory of Veterinary Etiological Biology, Key Laboratory of Veterinary Parasitology of Gansu Province, Lanzhou Veterinary Research Institute, Chinese Academy of Agricultural Sciences, Lanzhou 730046, China; zouyangdr@163.com; 5 College of Veterinary Medicine, Shanxi Agricultural University, Taigu, Jinzhong 030801, China

**Keywords:** dairy cattle, *Enterocytozoon bieneusi*, prevalence, zoonotic potential, Yunnan province, China

## Abstract

**Simple Summary:**

We first report the prevalence of *Enterocytozoon bieneusi* in Holstein Cows and dairy buffalo in Yunnan province of China, with a percentage of positivity of 0.59% (5/841). Two novel zoonotic potential genotypes of *E. bieneusi* were found. We analyzed the different influencing factors (region, season, variety, breeding mode, gender, age), but the differences were not statistically significant.

**Abstract:**

*Enterocytozoon bieneusi* is a fungus-like protist parasite that can cause diarrhea and enteric diseases. The infection of *E. bieneusi* has been reported in many host species, including cattle and humans. However, information on prevalence and genotype distribution of *E. bieneusi* in dairy cattle in Yunnan province in China is still absent. In this study, 490 Holstein Cows and 351 dairy buffalo fecal samples were collected from three regions in Yunnan province, China. By using nest-PCR that targets the internal transcribed spacer (ITS), we found that the prevalence of *E. bieneusi* was 0.59% (5/841). DNA sequence analysis showed that five *E. bieneusi* genotypes were identified in this study, including two novel genotypes, YNDCEB-90 and YNDCEB-174, and three known genotypes (I, J, BEB4). Phylogenetic analysis revealed that two novel genotypes, YNDCEB-90 and YNDCEB-174, were clustered into Group 1, representing the zoonotic potential. The remaining genotypes I, J, and BEB4, which are the most frequent genotypes of *E. bieneusi* infection in cattle and lead to *E. bieneusi* infection in humans, belonged to Group 2. Although the lower prevalence of *E. bieneusi* was detected in dairy cattle in Yunnan province, it indicates that dairy cattle should be considered to be one of the potential hosts for transmitting *E. bieneusi* to humans. These findings are important for the development of effective prevention strategies for microsporidiosis.

## 1. Introduction

*Enterocytozoon bieneusi* belongs to microsporidial species. It is an enteric unicellular microsporidian parasite that can infect invertebrate and vertebrate hosts worldwide [1]. The phylum microsporidia consists of more than 200 microsporidian genera with more than 1500 species [2,3]. *E. bieneusi* is the most prevalent pathogen in human beings and various mammals [4,5,6,7], causing symptomatic and asymptomatic intestinal infections through accidental ingestion of food or water that has been contaminated with the viable spores of *E. bieneusi*. *E. bieneusi* is also deemed as one of the riskiest opportunistic pathogens for patients with HIV/AIDS [8,9,10].

It is difficult to distinguish *E. bieneusi* from other microsporidia species by conventional staining methods due to the extremely small size of the spore. Currently, the most efficient method to characterize *E. bieneusi* is using PCR and DNA sequencing of the ribosomal internal transcribed spacer (ITS) [11]. The first cases of *E. bieneusi* infection in cattle were found in Germany, and two genotypes, I and J, were identified [12]. The prevalence of *E. bieneusi* in cattle ranged from 1.13% to 46.8% in China, the United States, Argentina, South Korea, Germany, Portugal, South Africa, Brazil, and the Czech Republic [12,13,14,15,16,17,18,19,20]. Although most of *E. bieneusi* genotypes are detected in cattle and belong to Group 2, these genotypes have also been identified in humans, animals, drinking water sources, and municipal sewage, indicating the possibility of cross-species or zoonotic *E. bieneusi* transmission [21,22].

Yunnan province has unique climatic characteristics, which include subtropical and tropical monsoon climates, and the minimum monthly average temperature is above 0 °C. This environment is suitable for the reproduction of intestinal protozoa, e.g., *E. bieneusi*. *E. bieneusi* affects not only the development of the livestock breeding industry, leading to the decline in production performance and loss of economic benefits, but it also pollutes water, soil, and food, causing food and waterborne outbreaks [23]. However, the information on prevalence and genotype distribution of *E. bieneusi* in dairy cattle in Yunnan province is still absent. In the present study, we use PCR and DNA sequencing to screen *E. bieneusi* infection in dairy cattle of Yunnan province and assess the risk factors for prevalence of *E. bieneusi,* such as geographic region, season, variety, breeding mode, gender, and age. The results of this study revealed that the infected animals can be identified as potential sources of *E. bieneusi* infection between dairy cattle and humans.

## 2. Materials and Methods

### 2.1. Collection of Specimens

Eight hundred forty-one fecal samples of dairy cattle were collected from June 2019 to August 2020 on 15 farms in Kunming city (*n* = 248), Dali city (*n* = 357), and Tengchong county (*n* = 236) in Yunnan province, China. The dairy cattle were divided into six groups: region group, season group, species group, breeding mode group, gender group, and age group. All the fecal samples were collected individually using sterile gloves and stored in a refrigerator at 4 °C until DNA extraction.

### 2.2. DNA Extraction and PCR Amplification

The 10 g of fresh fecal specimens of each dairy cattle was washed twice with distilled water to remove the impurities. Genomic DNA was extracted from 200 mg of fecal samples using an EZNAR stool DNA Kit (OMEGA, Biotek Inc. Norcross, GA, USA) according to the manufacturer’s recommendations. *E. bieneusi* infection was screened by nested PCR amplification of the ribosomal internal transcribed spacer (ITS) [24] and amplification of the loci MS1, MS3, MS4, and MS7 multilocus sequences to analyze the genotypes of *E. bieneusi*. Positive samples were further characterized by multilocus sequence typing analyses using loci MS1, MS3, MS4, and MS7 [25], and the amplification products were subjected to electrophoresis on 2% agarose gel to be observed.

### 2.3. Statistical Analysis

The P value, odds ratios (ORs), and their 95% confidence intervals (95%CIs) of variables, including region, season, variety, breeding model, gender, and age, were calculated using SPSS20.0 (IBM Corporation, Armonk, NY, USA) and SAS9.1 (SAS Institute Inc., Cary, NC, USA).

### 2.4. Sequencing and Phylogeny

The positive-PCR products were sequenced by Sangon Biotech (Kunming, China). The sequencing accuracy was confirmed by using two-directional sequencing. All the obtained sequences were aligned to the reference sequences available from the GenBank database using the Basic Local Alignment Search Tool (BLAST) (http://www.ncbi.nlm.nih.gov/BLAST accessed on 17 January 2021), and computer program DNAMAN 6.0 (Lynnon Biosoft, San Ramon, CA, USA) was used to identify the genotypes of *E. bieneusi*. The phylogenetic tree was constructed for assessing the genetic relationship between the *E. bieneusi* genotypes using the Neighbor-joining (NJ) method with MEGA5 (MEGA, Auckland City, New Zealand). The reliability analysis of the evolutionary tree was estimated by the Bootstrap test, repeated 1000 times, and more than 95% was the threshold of significance. The novel ITS sequences of *E. bieneusi* isolates were submitted to GenBank with the accession numbers MZ229914.1 for YNDCEB-90 and MZ229915.1 for YNDCEB-174.

## 3. Results

In this study, the global positive rate of *E. bieneusi* infection was 0.59% (5/841). As shown in Table 1, the *E. bieneusi* positive rates of the fecal specimens of dairy cattle in Kunming, Dali, and Tengchong were 0.81% (2/248), 0.56% (2/357), and 0.42% (1/236), respectively. There was no significant difference among the three regions (*p* = 0.856 > 0.05). The global infection rate of *E. bieneusi* in female cattle was 0.67% (5/751, 95%CI = 0.08–1.25), while no infection was found in male cattle. We further investigated the infection ratio as a seasonal variation. As shown in Table 1, the infection rate was 1.04% (1/96, 95%CI = 0.00–3.07) in autumn, 0.67% (4/599, 95%CI = 0.02–1.32) in summer, and no *E. bieneusi* infection was detected in winter. No significant difference was found in both the gender group (*p* = 0.774 >0.05) and season group (*p* = 0.535 > 0.05). Of the 841 analyzed samples, Holstein cows (positive ratio 0.61%, 3/490) showed higher infection rates than dairy buffalo (positive ratio 0.57%, 2/351), and there was no significant difference between the two varieties. To investigate whether the infection was associated with breeding modes, the cattle were classed into captivity group and grazing group, respectively. As shown in Table 1, the cattle raised in captivity showed an infection ratio of 1.44% (2/139, 95%CI = 0.00–3.42) while the cattle raised in grazing showed an infection ratio of 0.43% (3/702, 95%CI = 0.00–0.91). Both of the two breeding modes showed no significant difference (*p* = 0.156 > 0.05).

**Figure 1:**
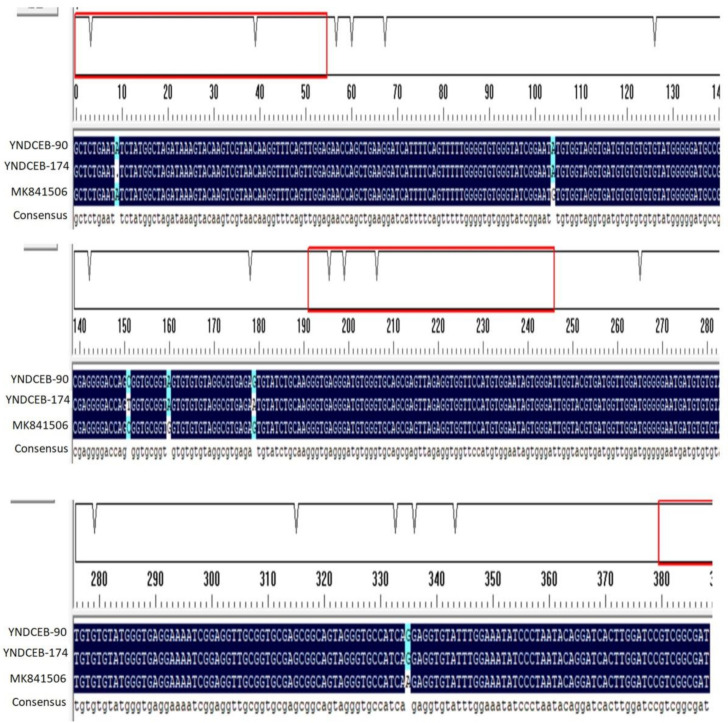
Sequence variation in the ITS region of the rRNA gene of *E. bieneusi* isolates. The ITS sequences of one known genotype MK841506 (genotype J) and two novel genotypes (YNDCEB-90 and YNDCEB-174) were identified in this study.

**Figure 2:**
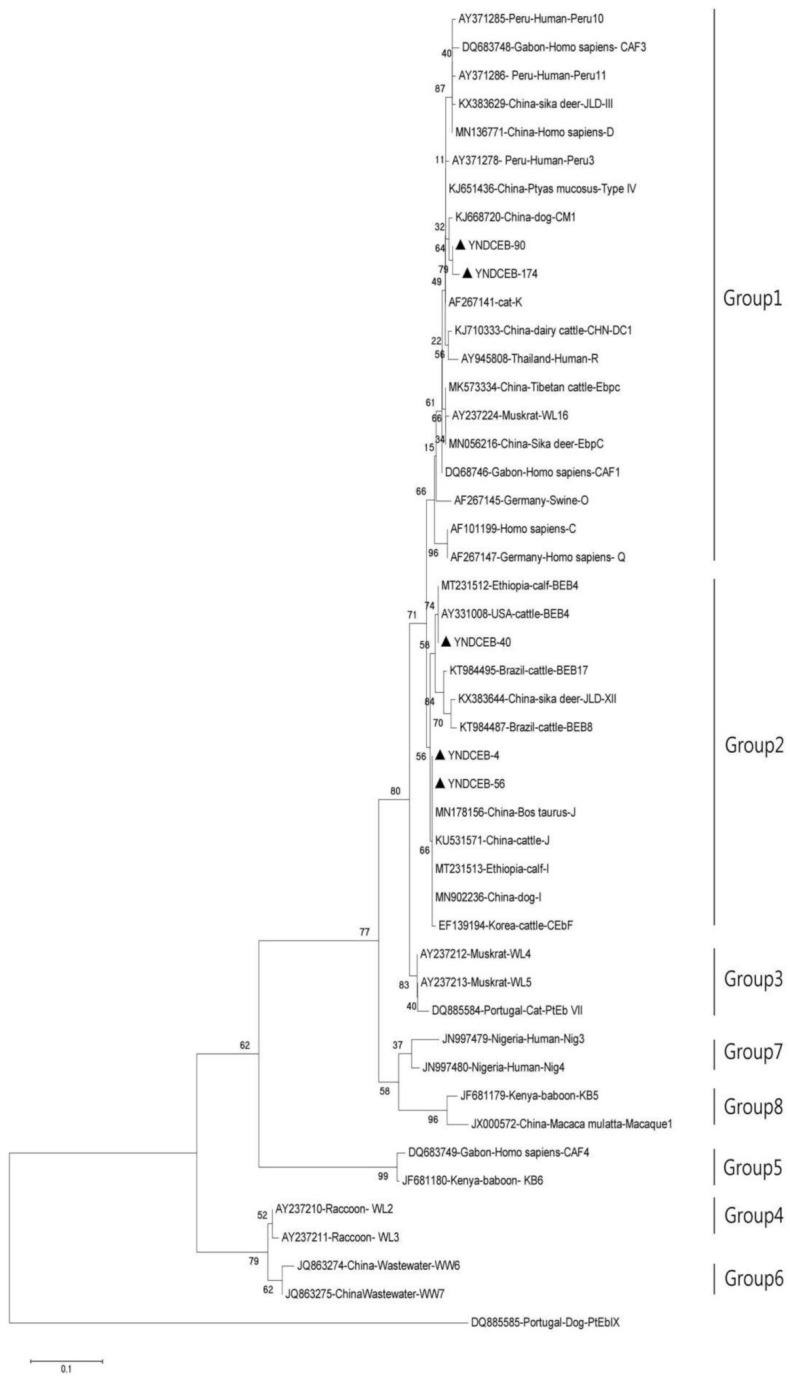
Phylogenetic relationships among *Enterocytozoon bieneusi* genotypes identified in this study and others already deposited in GenBank. Bootstrap values of >60% for 1000 replicates are shown on nodes. The known genotypes that were found around the world and the genotypes that were identified in this study are indicated with open and filled triangles, respectively.

The ITS ranges of positive samples were consistent with three known genotypes (I, J, BEB4) and two novel genotypes (YNDCEB-90, YNDCEB-174) which have 99.23% and 98.46% similarity with MK841506 (genotype J), respectively (Figure 1). Genotypes I and J were identified in Kunming. Genotypes BEB4 and YNDCEB-90 were identified in Dali. Genotype YNDCEB-174 was identified in Tengchong. The three known genotypes (I, J, and BEB4) were clustered into Group 2, and the two novel genotypes (YNDCEB-90, YNDCEB-174) were clustered into Group 1 (Figure 2). We identified three, zero, three, and zero types at the MS1, MS3, MS4, and MS7 loci, respectively.

## 4. Discussion

*E. bieneusi* is an important pathogen of neonatal calf diarrhea (NCD) that can cause high morbidity and mortality in dairy cattle [26]. While there is no vaccine commercially available to prevent *E. bieneusi* infection in humans and animals, effective measures to control *E. bieneusi* prevent the transmission of these parasite spores that pollute food, drinking water, and soil.

In this study, the overall prevalence of *E. bieneusi* in dairy cattle was 0.59% (5/841), which is lower than that of other provinces in China, such as Ningxia Hui autonomous region [13], Heilongjiang province [22], Henan province [27], Shaanxi province [28], Jiangsu province [29], Shandong province, Guangdong province, and Gansu province [30]. The different breeds, detection methods, geographical differences, and sample sizes are many factors that may contribute to the varying prevalence. Moreover, *E. bieneusi* was more likely to be found in autumn (odds ratio, OR = 1.56 [0.17, 14.16]) compared to summer, although the difference was not statistically significant (*p* > 0.05) (Table 1). Between the two breeds of cattle, the infection rate of *E. bieneusi* in Holstein cows was 0.61% (3/490), which was slightly higher than that of dairy buffalo 0.57% (2/351). The prevalence of *E. bieneusi* in captive cattle and free-ranging cattle did not differ significantly (*p* > 0.05); however, it was frequently higher (OR = 3.40 [0.56, 20.55]) in free-ranging cattle (Table 1). This result suggested that we should pay attention to strengthening the prevention of free-ranging cattle. Interestingly, in the present study, the pre- and post-weaned cattle were not found to be infected by *E. bieneusi*. The result was different in previous studies. We speculated that the difference could result from the better care that was provided to pre- and post-weaned cattle in our investigated area.

In this study, five ITS genotypes of *E. bieneusi* were identified in dairy cattle in Yunnan province, including three known genotypes (I, J, BEB4) and two novel genotypes (YNDCEB-90, YNDCEB-174). The genotypes I (*n* = 1) and J (*n* = 1) were most prevalent in dairy cattle in the present study, which were similar to results reported in Ningxia Hui autonomous region and Henan province [13], Heilongjiang province [21], northeastern China [22], Shaanxi province [27], Jiangsu province [29], Shandong province [31], and Jilin province [32] in China. The prevalence of *E. bieneusi* in Yunnan province was also similar to that in the United States [14], Argentina [15], and other countries. These findings revealed that genotypes I and J are prevalent worldwide. In this study, we found that YNDCEB-90 has three single-nucleotide substitutions (SNPs), and YNDCEB-174 has SNPs at the ITS region (243 bp) in Figure 1. The ITS sequences of the two novel genotypes (YNDCEB-90, YNDCEB-174) have 99.23% and 98.46% similarity with MK841506 (genotype J), respectively. The detection of two novel genotypes suggested the possibility of genetic variations in *E. bieneusi* in dairy cattle in Yunnan province. The Phylogenetic tree analysis showed that two novel genotypes were classed into the category previously described as a zoonotic Group 1, and the three known genotypes (I, J, and BEB4) were clustered into Group 2 (Figure 2). All of those genotypes have been detected in humans [21]. Therefore, the dairy cattle may be potential hosts of human-pathogenic *E. bieneusi.* In this study, we only identified three, zero, three, and zero types at the MS1, MS3, MS4, and MS7 loci, respectively. The result may be that there was only a small amount of *E. bieneusi* in the fecal specimen, and the different PCR primers have different amplification efficiencies, leading to the identification of zero types at MS3 and MS7 loci.

## 5. Conclusions

The present study is the first report of the prevalence (0.59%, 5/841) of *E. bieneusi* infection in dairy cattle in Yunnan province, China. We demonstrated the three known ITS genotypes (I, J, BEB4) and two novel genotypes (YNDCEB-90, YNDCEB-174) that have zoonotic potential. The different influencing factors (such as region, season, variety, breeding mode, gender, and age) were not statistically significant. Since there is a deficiency of effective prophylactic and therapeutic strategies for *E. bieneusi*, and due to the existing threat posed by the fecal–oral transmission of *E. bieneusi*, molecular epidemiological investigation is required to be conducted in more animal hosts.

## Figures and Tables

**Table 1:** The infection of *E. bieneusi* in some dairy cattle in Yunnan Province.

Factors	Category	Number Tested	Number Positive	Prevalence (%) [95%CI]	Genotype	OR (95%, CI)	*p*-Value
Region	Kunming	248	2	0.81 [0.00–1.92]	I, J	1.91 (0.17–21.21)	0.856
	Dali	357	2	0.56 [0.00–1.33]	BEB4, YNDCEB-90	1.32 (0.12–14.68)	
	Tengchong	236	1	0.42 [0.00–1.25]	YNDCEB-174	Reference	
Season	Summer	599	4	0.67 [0.02–1.32]	I, J, YNDCEB-90, YNDCEB-174	Reference	0.535
	Autumn	96	1	1.04 [0.00–3.07]	BEB4 (1)	1.56 (0.17–14.16)	
	Winter	146	0	0	0	-	
Variety	Holstein cows	490	3	0.61 [0.00–1.30]	I, J, BEB4	1.07 (0.18–6.47)	0.937
	Dairy buffalo	351	2	0.57 [0.00–1.36]	YNDCEB-90, YNDCEB-174	Reference	
Breeding Mode	Captive	702	3	0.43 [0.00–0.91]	I, J, BEB4	Reference	0.156
	Free-ranging	139	2	1.44 [0.00–3.42]	YNDCEB-90, YNDCEB-174	3.40 (0.56–20.55)	
Gender	Male	90	0	0	-	-	0.438
	Female	751	5	0.67 [0.08–1.25]	I, J, BEB4, YNDCEB-90,YNDCEB-174	-	
Age	Pre-weaned cattle (0–2 month)	18	0	0	-	-	0.774
	Post-weaned cattle (3–6 month)	42	0	0	-	-	
	Growing cattle (7 months to 1.5 years)	73	1	1.37 [0.00–4.04]	YNDCEB-90	2.44 (0.27–22.17)	
	Adult cattle (>1.5 years)	708	4	0.56 [0.01–1.12]	I, J, BEB4, YNDCEB-174	Reference	
Total		841	5	0.59	I, J, BEB4, YNDCEB-90 YNDCEB-174	-	-

Notes: OR, odds ratio; CI, confidence interval; Reference, minimum infection rate.

## Data Availability

Data are contained within the article.
